# Efficacy of a Computerized Therapeutic Decision-Making Algorithm in a Fracture Liaison Service Targeting Hip Fracture Patients

**DOI:** 10.3390/jcm14197062

**Published:** 2025-10-06

**Authors:** Rachel Chava Rosenblum, Arthur Kogan, Dana Herzberg, Maysara Najjar, Oded Hershkovich, Orit Twito, Raphael Lotan

**Affiliations:** 1 Endocrinology Unit, Wolfson Medical Center, Holon 58100, Israel; rachrose613@gmail.com (R.C.R.); oritt@wmc.gov.il (O.T.); 2 The Gray Faculty of Medical and Health Sciences, Tel Aviv University, Tel Aviv 69978, Israel; dr.lotan@gmail.com; 3 Orthopedic Department, Wolfson Medical Center, Holon 58100, Israel

**Keywords:** osteoporosis, hip fracture, fracture liaison service, Vitamin D

## Abstract

**Introduction:** This study evaluates the efficacy of a nurse practitioner-managed, computer algorithm-supported institutional fracture liaison service (FLS) that provides treatment recommendations for patients with hip fractures. **Methods:** A retrospective study included patients hospitalized in the Orthopedic ward with hip fractures between April 1 and October 31. The decision-making algorithm recommends zoledronic acid as the default medication, except for patients younger than 65 years, with estimated glomerular filtration rate (eGFR) <35 or prior osteoporosis therapy, who are ordered to undergo endocrinology consultation. Patients with vitamin D deficiency/insufficiency are given a loading dose. **Results:** Two hundred and eight hip fracture patients were identified. The cohort was predominantly female (137/208, 65.9%); the mean age was 79.9 ± 9.6 years. Nurse practitioner evaluation was performed in 200/208 patients (96.2%). The algorithm provided a treatment recommendation in 140 out of 200 (70.0%), while 60 out of 200 (30.0%) required an endocrinology consultation. A Vitamin D loading dose was given in 89/99 (89.9%) deficiency and 44/62 (71.0%) insufficiency cases. **Conclusions:** This simplified algorithm-based FLS model demonstrated practicality and feasibility in providing therapeutic recommendations with minimal physician intervention.

## 1. Introduction

The lifetime risk for fragility fracture is approximately 50% in women and 20% in men over 50 years of age [1,2]. The risk of osteoporotic fracture is doubled in patients with a history of prior osteoporotic fractures [3]. In an extensive US database, the incidence of a second fracture after a hip fracture was 22–25.5% within the first post-fracture year; the second fracture was most likely of the same type as the incident fracture [4]. Osteoporotic fractures have significant effects on morbidity and quality of life and have been shown to increase mortality risk [5]. Furthermore, they have substantial economic ramifications [4].

Despite the high risk for additional fractures, osteoporosis treatment rates in patients after osteoporotic fracture are dismal, remaining at approximately 20% [6]. Fracture Liaison Services (FLS) were initiated in the 1990s [7,8] and brought into international use in 2012 with the launch of the pivotal International Osteoporosis Foundation (IOF) global ‘Capture the Fracture’ campaign [9]. The IOF Capture the Fracture Best Practice Framework (BPF) provides criteria for recognition and rating of successful FLS worldwide [10]. The campaign aims to close the secondary fracture prevention treatment ‘gap’ and reduce the risk of future fractures. Various FLS models exist, and intensive services incorporating patient identification, assessment, and treatment tend to deliver the best outcomes [11]. FLS programs require the collaboration of healthcare workers within a multidisciplinary team and the implementation of meticulously planned protocols with multiple phases [12]. Observational studies have shown an association between the establishment of FLS programs and an increase in antiresorptive therapy rates, as well as a reduction in fracture and mortality rates within 12 months of an incident fracture [13]. Hundreds of FLS programs exist in 55 countries; however, the map of Best Practices indicates a dearth of programs in resource-poor regions throughout the developing world [14].

Therapeutic decision-making for hip fracture patients in the FLS setting requires consideration of drug efficacy, economic factors, drug contraindications, side effects and potential risks. Drugs that have demonstrated a reduction in hip fracture rates in randomized controlled trials include alendronate, risedronate, zoledronic acid, denosumab, and Romosozumab [15,16,17,18,19]. Meta-analyses have also demonstrated a reduction in hip fractures with teriparatide [20]. However, head-to-head osteoporosis drug trials with fracture endpoints are scarce [19,20]. In secondary fracture prevention, the initiation of osteoporosis treatment has been associated with lower recurrent fracture rates [21]. The HORIZON recurrent fracture trial demonstrated a reduction in recurrent fractures and mortality in patients after hip fractures treated with Zoledronic acid [22]. The American Society for Bone and Mineral Research (ASBMR) and the Center for Medical Technology Policy (CMTP) multistakeholder coalition consensus clinical recommendations for secondary fracture prevention recommend oral bisphosphonates as first-line therapy for such patients and intravenous zoledronate or denosumab if oral bisphosphonates pose difficulties; while anabolic agents are recommended for patients with high risk [23]. Our National Health Basket authorizes first-line osteoporosis treatment with zoledronic acid or denosumab after hip fracture [24]. While denosumab is not incorporated into the bone matrix and must be administered every 6 months, the antiresorptive effects of a single 5 mg IV dose of zoledronate are sustained for 3 years [25].

Specific relevant clinical and laboratory scenarios should be considered in therapeutic decision-making. For instance, the prevalence of secondary osteoporosis is higher in younger patients, accounting for more than 50% of cases in premenopausal women; therefore, more extensive investigations are necessary in young patients with osteoporosis [26]. Furthermore, fractures that occur despite osteoporosis therapy may allude to treatment failure and raise considerations for changing therapy [27]. Patients with chronic kidney disease pose unique therapeutic dilemmas due to the coexistence of varied pathophysiological forms of chronic kidney disease-related mineral bone disease [28], estimated glomerular filtration rate (eGFR) cutoffs of the various therapies, and risk of hypocalcemia after anti-resorptive treatment. Pre-treatment Vitamin D levels are necessary as vitamin D deficiency is associated with increased mortality and complications as well as reduced post-surgical functional recovery in hip fracture patients [29]. Additionally, patients in HORIZON with vitamin D deficiency developed hypocalcemia after receiving zoledronic acid treatment [15]; therefore, patients in the HORIZON recurrent fracture trial with vitamin D deficiency were given a loading dose of vitamin D before receiving zoledronic acid therapy [22].

This study evaluated the functionality of a low-cost, low-resource, streamlined, computerized, algorithm-supported, Nurse Practitioner-managed institutional FLS model.

## 2. Methods

This is a retrospective study evaluating the functionality of the computerized algorithm for FLS in patients admitted to the Orthopedic Department with hip fractures between 1 April and 31 October 2024, identified by a dedicated Nurse Practitioner.

**Figure 1:**
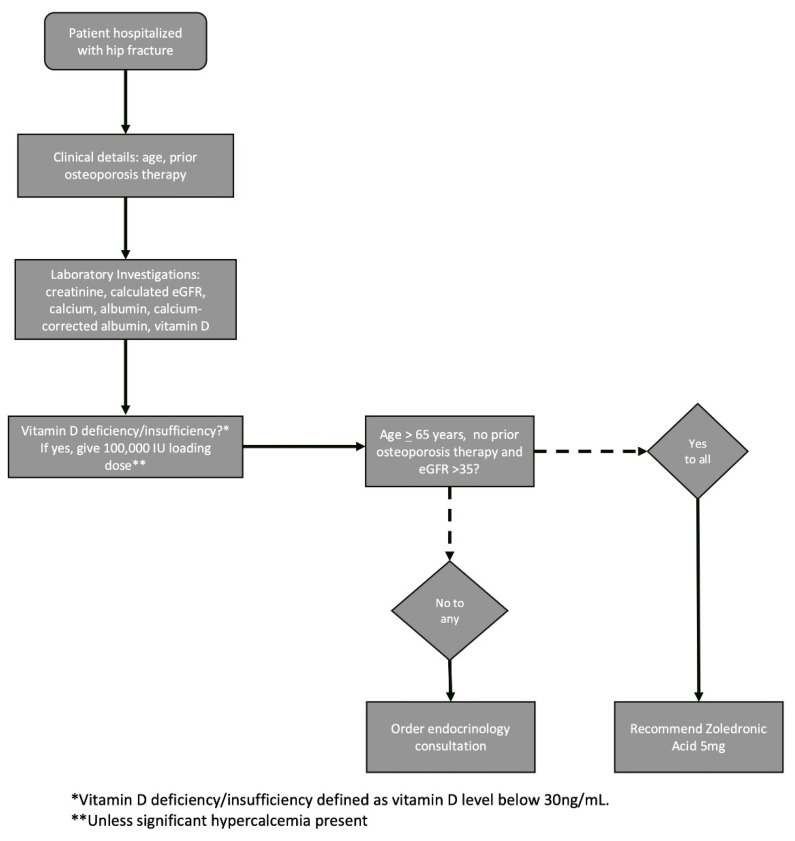
FLS patient work-up and treatment algorithm.

Osteoporosis therapy recommendations were provided based on a decision-making algorithm developed by the endocrinology team. Zoledronic acid was chosen as the algorithm’s default therapy recommendation due to the drug’s authorization as a first-line therapy for hip fracture in the Israeli Health Basket, its demonstrated mortality benefit in this setting, and its long-lasting activity. Denosumab, which can also be given as a first-line treatment after hip fracture in Israel, was not chosen as the protocol standard due to the importance of continued adherence with this drug and the risks associated with its discontinuation. The therapeutic decision-making algorithm (Figure 1) requires only three inputs: patient age, current eGFR and use of prior osteoporosis therapy. For treatment-naïve patients aged 65 years and above with eGFR > 35, zoledronic acid is recommended. An endocrinology consultation was recommended for patients who did not fit these criteria to provide an individualized treatment decision.

The described FLS protocol was integrated as a computerized component into the electronic medical records (EMR). The Orthopedic Department’s dedicated nurse practitioner was responsible for identifying patients, running the algorithm, and ordering endocrinology consultations as necessary.

Exclusion criteria included patients with a high-energy fracture mechanism or non-proximal femoral fracture sites, including subtrochanteric, distal or unknown fracture sites.

In-hospital laboratory assessment included serum creatinine, calcium, albumin, and vitamin D levels. Laboratory assays used were creatinine testing by kinetic colorimetric assay (CREJ2, Jaffe Gen.2, Cobas), calcium testing by photometric assay (CA2, Cobas, Basel, Switzerland), albumin testing by colorimetric assay (ALB2, Cobas), and total 25-hydroxyvitamin D by electrochemiluminescence immunoassay (Elecsys Vitamin D total III, Cobas), all by Roche Diagnostics. eGFR was estimated by the hospital laboratory using the Modification of Diet in Renal Disease (MDRD) formula, and in cases where the calculated eGFR was above 120, it was corrected to 120. Serum calcium was corrected for albumin using the formula: corrected calcium = 0.8 × (4-patient albumin) + serum calcium. Hypercalcemia was defined as a corrected calcium level above the upper limit of normal and was considered mild if the corrected calcium level was below 12 mg/dL. Vitamin D deficiency was defined as a level below 20 ng/mL, and insufficiency was defined as a level between 20 and <30 ng/mL. Hypoalbuminemia was defined as a serum albumin level <3.5 g/dL, and clinically significant hypoalbuminemia as <2.5 g/dL. Patients with vitamin D deficiency/insufficiency were given a loading dose of 100,000 units during hospitalization as long as hypercalcemia was not present. Endocrinology consultation was advised for all patients with hypercalcemia to consider in-hospital therapy and evaluate for relevant causes of secondary osteoporosis.

At discharge, patients were recommended a high-calcium diet and continued vitamin D treatment at a dose of 1000 IU per day. The algorithm treatment recommendation or endocrinology consultation appeared in the hospital discharge letter. Patients could then receive the recommended osteoporosis therapy in the treating rehabilitation department or in the outpatient setting. After discharge, patients received telephone communications from the FLS program coordinator to remind them of the importance of continuing this therapy and to assist with any related difficulties. Patients who required further investigation were followed up at the outpatient clinic in the hospital’s endocrinology department.

Patient data were extracted from institutional electronic medical records, including age, sex, fracture date, fracture subtype, osteoporosis therapy before fracture, hospital laboratory results, use of the FLS treatment algorithm, endocrinology consultation recommendations, and administration of a vitamin D loading dose.

The Institutional Ethics Committee approved the study (0213-21-WOMC). Following Helsinki regulations regarding clinical studies based on chart review, informed consent was waived.

Statistical analyses were performed using Microsoft Excel for Mac version 16.66.1. Categorical variables are presented as counts and percentages. In contrast, continuous variables are presented as mean ± standard deviation (SD) for normally distributed variables or as the median and interquartile range (IQR) where appropriate. Differences between groups were assessed using the independent samples t-test for continuous variables and Fisher’s exact test for categorical variables. A two-tailed *p*-value <0.05 was considered statistically significant.

## 3. Results

**Figure 2:**
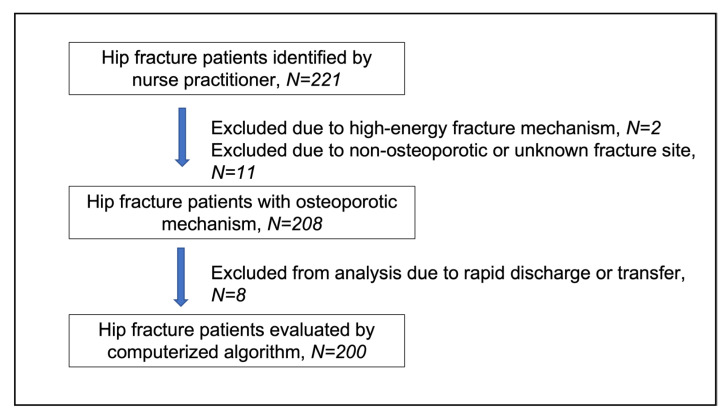
Flow-chart for patient evaluation.

Two hundred and twenty-two hospitalizations with hip fractures were identified in 221 patients by the nurse practitioner within the specified time frame. Of these, 13 were excluded from the study due to high-energy fracture mechanism (n = 2), subtrochanteric or distal femoral fracture site (n = 10), and unspecified fracture site (n = 1). Therefore, a cohort of 208 patients with osteoporotic hip fractures was included in the analysis. A study flow-chart is shown in Figure 2.

**Figure 3:**
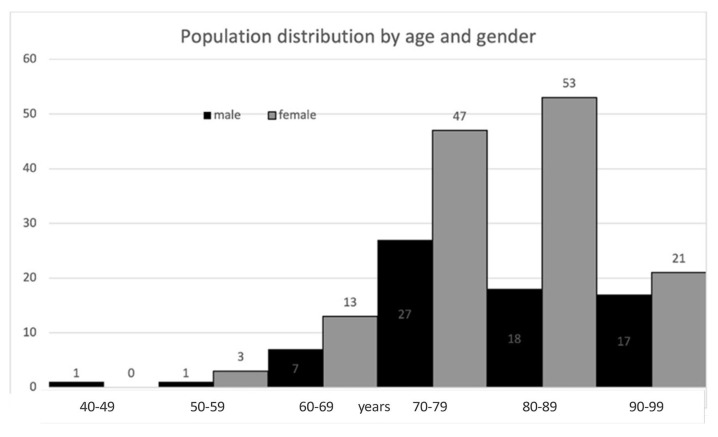
Population distribution of the FLS cohort.

The cohort consisted chiefly of elderly patients and was predominantly female. Baseline characteristics are described in Table 1, and age and gender distribution are depicted in Figure 3. Twelve fracture cases (5.8%) were diagnosed below age 65 years; 8 of these patients (66.7%) had at least one documented risk factor or secondary cause of osteoporosis, including malignancy with bone metastases (n = 1), spinal muscular atrophy (n = 1), primary hyperparathyroidism (n = 1), diabetes mellitus (n = 1), epilepsy (n = 1), premature ovarian insufficiency (n = 1), family history of osteoporosis (n = 2), smoking (n = 2), drug abuse (n = 1), and hepatitis C virus infection (n = 1).

**Figure 4:**
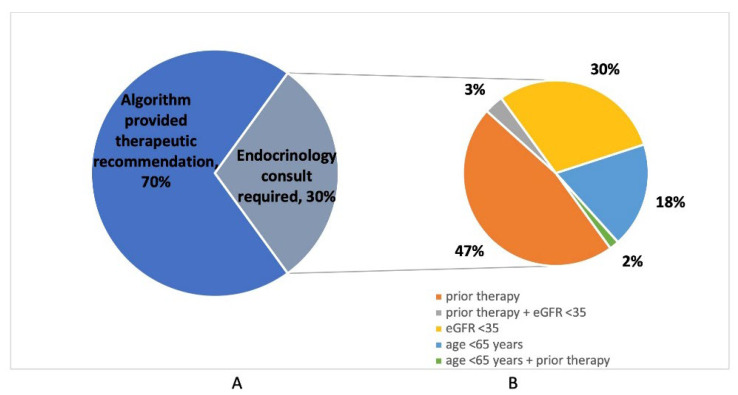
Performance of the Therapeutic Decision-making Algorithm. (**A**) Recommendations made by the decision-making tool. (**B**) Reasons for Endocrinology consultation.

Nurse Practitioner assessments using a computerized algorithm were performed in 200 out of 208 osteoporotic fracture cases (96.2%); cases were missed due to rapid patient discharge or transfer. Of those assessed, the computerized FLS algorithm provided a direct recommendation for zoledronic acid in 140/200 (70.0%) of cases, while 60/200 (30.0%) required an endocrinology consultation (Table 2). Reasons for requests for endocrinology consultation included known or suspected previous osteoporosis therapy in 31/60 (51.7%), eGFR <35 in 20/60 (33.3%), and age below 65 years in 12/60 (20.0%) of patients (Figure 4). Previous osteoporosis therapies included oral bisphosphonates (n = 20), zoledronic acid (n = 2), denosumab (n = 4), and teriparatide (n = 2); a further three patients were suspected of having received previous therapy, but additional details were unknown.

Hypercalcemia was noted in 4/208 patients (1.9%); all cases were mild. Hypoalbuminemia was noted in 68/208 cases (32.7%); only one case was clinically significant. Vitamin D deficiency and insufficiency accounted for 79.7% (161/202) of the cohort with known vitamin D levels. Vitamin D loading doses were given in 89/99 (89.9%) of deficiency and 44/62 (71.0%) of insufficiency cases (Table 3).

An endocrinology consultation was performed in 56 out of 60 requested cases (93.3%); Table 2 describes the therapies recommended during the consultation. For patients with a history of prior osteoporosis therapy, treatment recommendations included zoledronic acid (n = 17), denosumab (n = 2), and further investigations (n = 8); consultation was not performed in one case. For those with an eGFR <35, treatment recommendations included zoledronic acid (n = 1), further investigations (n = 7), and no therapy (n = 8); consultation was not performed in two cases. For patients under 65, recommendations included zoledronic acid (n = 3) and further investigations (n = 7); consultation was not performed in one case. One young patient with prior treatment was recommended for teriparatide treatment. Two patients with low eGFR and a history of prior treatment were recommended denosumab and further investigations, respectively. Further investigations were requested in cases where anabolic therapy was considered, eGFR was low, or patients were young, and secondary osteoporosis was suspected.

Comparing the clinical and laboratory characteristics of patients who received treatment recommendations via the computerized FLS algorithm and those requiring endocrinology consultation, the latter were more likely to be female and have a lower eGFR (Table 4).

## 4. Discussion

Numerous FLS programs exist worldwide and are implemented in varying performance models tailored to fit the structure of local or regional healthcare systems. Straightforward approaches that require minimal clinical and laboratory data, as well as minimal physician intervention, may encourage the implementation of such programs in resource-constrained institutions and regions. We developed an FLS model involving a simple flowchart, an EMR-integrated algorithm-based decision-making tool which a nurse practitioner could perform.

In this FLS model, 96.2% of patients identified by the Nurse Practitioner were evaluated during admission to the Orthopedic Department. Prior osteoporosis medications were reviewed in all tracked cases. The application of the algorithm generated a direct therapeutic recommendation in 70% of cases, while an endocrinology consultation was required in only 30%. The most frequent reason for endocrinology consultation was prior osteoporosis therapy, accounting for over 50% of consultations; almost all prior treatments were bisphosphonates, as would be expected in an elderly hip fracture cohort. Anabolic therapies were considered for several of these patients; however, further investigations were requested in most cases to allow individualized decision-making. A third of consultations were due to low eGFR, alluding to the presence of chronic kidney disease-related metabolic bone disease. This complex phenomenon raises multiple questions, including the underlying pathophysiology of the individual case, the current and predicted eGFR of the patient, and the ability to discontinue therapy in this context if necessary. Ultimately, while some of these patients were recommended denosumab, in other cases, no treatment was recommended. Only a few consultations were due to young patient age; most of these patients had documented risk factors or secondary causes of osteoporosis, and the others were recommended to undergo extensive evaluation.

Overall, therapeutic recommendations for osteoporosis were provided during hospitalization in 165/200 (82.5%) of the evaluated patients; further investigations were recommended in 23/200 (11.5%), and no therapy was recommended in 8/200 (4.0%). Implementation of treatment recommendations was followed up post-discharge by the FLS coordinator.

Vitamin D deficiency or insufficiency was noted in the vast majority (almost 80%) of the cohort, alluding to the increased mortality and complication risk in this cohort, [29], as well as an increased risk of hypocalcemia after zoledronic acid treatment, if left untreated [15]. Over 80% of patients with insufficiency or deficiency were given an in-hospital loading dose of vitamin D, thereby enabling the early provision of osteoporosis therapy post-discharge with a reduced risk of hypocalcemia. A loading dose was not administered if hypercalcemia was present or if the vitamin D level was unavailable at the time of the nurse practitioner evaluation.

This study has several limitations. A major limitation is its retrospective, single-center nature, which may limit the generalizability of the findings to other healthcare systems, particularly those with different reimbursement structures or scope-of-practice regulations for nurse practitioners. Second, the study did not assess post-discharge treatment initiation, adherence, or long-term outcomes such as fracture recurrence or mortality. These data are essential for evaluating the actual clinical impact of the FLS model and are planned for future analysis. Third, the use of the simple decision-making algorithm eliminates the element of personalized medicine in therapeutic decision-making. The decision-making algorithm prioritized zoledronic acid as the default therapy, in line with national health policy, which may have limited the use of other therapies with higher rates of fracture prevention, including anabolic therapies and denosumab. These were not included as first-line recommendations due to cost and policy constraints for anabolic therapies, as well as adherence concerns for denosumab. The use of zoledronic acid as a first-line treatment may not reflect the optimal treatment strategy in all cases. Ultimately, anabolic therapy was recommended in very few cases in our study. Finally, the reliance on nurse practitioners has its drawbacks, including the reduced involvement of treating physicians in osteoporosis care and limited authorizations granted to nurse practitioners. The model’s applicability may also be limited in healthcare settings where electronic medical record integration is not feasible.

The study group aims to facilitate the implementation of a streamlined, computerized EMR algorithm-based FLS model in other medical institutions across Israel. The development of the algorithm as an automated component in the Chameleon EMR program, utilized by many Israeli medical institutions, makes this a feasible goal. We further propose that this simple, low-cost FLS model be adopted by other institutions worldwide, particularly those with limited resources. A crucial potential improvement to the model would be broadening its scope to include other types of osteoporotic fractures; however, this would necessitate the development of fracture-specific, appropriate treatment algorithms.

In conclusion, this low-cost, low-resource nurse-practitioner-mediated, computerized algorithm-based FLS model provided osteoporosis treatment recommendations for most hip fracture patients, demonstrating its potential in providing therapeutic recommendations to large cohorts of hip fracture patients in settings with limited resources. Large, prospective multi-center studies are necessary to determine the value of the model in a broader clinical setting.

## Figures and Tables

**Table 1:** Baseline Characteristics of 208 hip fracture patients.

Clinical Characteristics	
Age (years)	79.9 ± 9.6 (42–98)
Gender	
Male	71/208 (34.1%)
Female	137/208 (65.9%)
Fracture type	
Subcapital	102/208 (49.0%)
Pertrochanteric	105/208 (50.5%)
Intertrochanteric	1/208 (0.5%)
Prior osteoporosis therapy	
Yes	28/208 (13.5%)
No	172/208 (82.7%)
Unknown **	8/208 (3.8%)
Laboratory findings	
Estimated GFR (mL/min)	66.2 ± 25.3 (10–120) *
Serum albumin level (g/dL)	3.8 ± 0.5
Corrected calcium level (mg/dL)	9.3 ± 0.5
Vitamin D level (ng/mL)	21.6 ± 11.7 (n = 202)

Continuous variables are presented as mean ± standard deviation, (range). * Estimated eGFR was calculated as above 120 in 7 cases and corrected to 120. ** Of the cases described here as unknown, three were suspected of having had prior therapy, but details were unavailable.

**Table 2:** Performance of the therapeutic decision-making algorithm in provision of osteoporosis treatment recommendations.

Algorithm Results	
Algorithm provided treatment recommendation	140/200 (70.0%)
Algorithm recommended endocrinology consultation	60/200 (30.0%)
Reason for endocrinology consultation	
Patient age <65 years	12/60 * (20.0%)
eGFR <35	20/60 ** (33.3%)
Prior osteoporosis therapy ***	31/60 (51.7%)
Treatment recommendations per endocrinology consultation	
Zoledronic acid	21/60 (35.0%)
Denosumab	3/60 (5.0%)
Teriparatide	1/60 (1.7%)
Further investigations	23/60 (38.3%)
Drug therapy not recommended	8/60 (13.3%)
Endocrinology consultation not performed	4/60 (6.7%)

* One patient was younger than 65 years and had prior osteoporosis therapy. ** Two patients had prior osteoporosis therapy and eGFR <35. *** Three patients had suspected prior therapy without further details.

**Table 3:** Baseline Vitamin D levels and administration of a loading dose.

Baseline Vitamin D Level	Proportion of Patients (n = 202) *	Loading Dose Given (n = 133)
Deficiency (<20 ng/mL)	99/202(49.0%)	89/99 (89.9%)
Insufficiency (20–<30 ng/mL)	62/202 (30.7%)	44/62 (71.0%)
Sufficiency (>30 ng/mL)	41/202 (20.3%)	0

* Vitamin D levels were unknown in 6/208 patients (2.9%).

**Table 4:** Characteristics of patients given therapeutic recommendations by a computerized FLS algorithm and patients requiring endocrinology consultation.

	Algorithm Treated (n = 140)	Endocrinology Consultation Required (n = 60)	*p*-Value
Age (years)	81.0 ± 8.4	77.8 ± 12.2	0.0669
Gender			
Male	54/68 (79.4%)	14/68 (20.6%)	0.0147
Female	86/132 (65.6%)	46/132 (34.8%)	
Fracture type			
Subcapital	68/97 (70.1%)	29/97 (29.9%)	0.1229
Pertrochanteric	72/102 (70.6%)	30/102 (29.4%)	
Estimated GFR (mL/min)	69.7 ± 21.5	58.8 ± 31.9	0.0168
Albumin level (g/dL	3.8 ± 0.5	3.7 ± 0.6	0.5361
Calcium level (mg/dL)	9.3 ± 0.5	9.3 ± 0.5	0.5052
Vitamin D level (ng/mL)	20.9 ± 11.7	22.9 ± 12.1	0.2812

## Data Availability

The data presented in this study are available on request from the corresponding author.
